# Personal

**Published:** 1911-07

**Authors:** 


### PERSONAL

---

One of the important surgical papers presented at the recent annual meeting of the Medical Society of the State of New York, held at Albany, was one read by Dr. Edward R. McGuire, of Buffalo, on Cerebral Compression. Its importance as a contribution to surgical literature cannot be overestimated and it takes its place as one of the most valuable. It is published elsewhere in the Journal.

---

Dr. George William Seitz, of Buffalo, announces the removal of his office and residence from 225 East Genesee Street to 1137 Ellicott Street, near Riley. Practice limited to diseases of the nose, throat and ear.

---

Dr. Augustus S. Downing, of Albany, first assistant commissioner, New York state education department, was one of the speakers at the banquet of the alumni of the department of pharmacy, University of Buffalo, held at the Statler Hotel, June 1.

---

Mr. Charles F. Wheelock, formerly chief of the examinations division of the New York state department of education, Albany, has been promoted to the position of second assistant commissioner of education. The new chief of the examinations division is Mr. Harlan H. Horner, to whom all requests or information regarding state license examinations should be addressed.

---

Dr. Herman D. Andrews, of Buffalo, announces the removal of his offices from 399 Delaware Avenue to his residence 486 Franklin Street, near Allen. Hours: 9 A.M., 1 P.M. and by appointment. Telephone, Tupper 743.

---

Dr. Thomas H. McKee, of Buffalo, delivered the address to the graduates of the nurses' training class of the Woman's Christian Association Hospital, Jamestown, on Thursday evening, June 1. The members of the class consisted of nine young women. Mrs. Frank E. Gifford, daughter of the late Gov. Reuben E. Fenton, presented the diplomas.

Dr. F. Park Lewis, of Buffalo, attended the meeting of the American Medical Association at Los Angeles, June 27-30, where he presented the Report on Blindness. He will also attend the meeting of the National Education Association at San Francisco, July 8-15, where he will present a report on The Conservation of Vision. After returning to Buffalo he will sail on the La Loraine to join his family in France. Dr. Lewis will return to Buffalo about the middle of September.

---

Dr. Roswell Park, of Buffalo, attended the recent annual meeting and banquet of the Ontario Medical Association held at Niagara Falls, Ont. By invitation he read a paper entitled, "Remarks on the Early History of Medicine in America," which is published elsewhere in the Journal.

---

Dr. and Mrs. Allen A. Jones, of Buffalo, are enjoying a trip through the state of California.

---

Health Commissioner Fronczak, of Buffalo, attended the conference of the National Housing Association in New York, in June, representing Buffalo.

---

Professor E. Fuch, of Vienna, whose reputation as a teacher and author in ophthalmology is international, comes to this country this year as the guest of the American Ophthalmological Society and will contribute papers to the program of its meeting at New London, Conn., on July 11 and 12. It is an occasion of unusual interest by reason of the participation of so distinguished a colleague.

---

Dr. William F. Gallivan, of Buffalo, a graduate of the University of Buffalo, class of 1909, has been appointed resident physician and instructor in the new maternity school of Bellevue Hospital, New York.

---

Dr. Irving M. Snow, of Buffalo, sailed in June to spend three months in Germany, combining recreation with study.

---

Dr. Alfred E. Diehl, of Buffalo, has removed from 362 Pearl Street to 448 Delaware Avenue. Hours: 9 to 1, 7 to 8, P.M. Telephone, Tupper 592.

---

Drs. Herman E. Hayd and W. Scott Renner, of Buffalo, attended the reunion of the medical graduates of McGill University held at Montreal, June 5. Dr. Hayd is an alumnus of the university, 1881, and Dr. Renner also, class of 1884.

Chancellor Charles P. Norton of the University of Buffalo sailed recently for a two months' tour through Europe.

---

Dr. George B. Young of Chicago, has been appointed by the Mayor of that city, health commissioner, to succeed Dr. William A. Evans. Dr. Young has an enviable record to live up to but he probably will find no difficulty in handling the problems connected with this office. Dr. Young, for six years, was in charge of the Marine Hospital in Chicago.

